# Probiotics Prevent Intestinal Barrier Dysfunction in Acute Pancreatitis in Rats via Induction of Ileal Mucosal Glutathione Biosynthesis

**DOI:** 10.1371/journal.pone.0004512

**Published:** 2009-02-18

**Authors:** Femke Lutgendorff, Rian M. Nijmeijer, Per A. Sandström, Lena M. Trulsson, Karl-Eric Magnusson, Harro M. Timmerman, L. Paul van Minnen, Ger T. Rijkers, Hein G. Gooszen, Louis M. A. Akkermans, Johan D. Söderholm

**Affiliations:** 1 Department of Clinical and Experimental Medicine, Division of Surgery, Linköping University, Linköping, Sweden; 2 Gastrointestinal Research Unit, Department of Surgery, University Medical Center, Utrecht, the Netherlands; 3 Department of Clinical and Experimental Medicine, Division of Medical Microbiology, Linköping University, Linköping, Sweden; University of Hong Kong, Hong Kong

## Abstract

**Background:**

During acute pancreatitis (AP), oxidative stress contributes to intestinal barrier failure. We studied actions of multispecies probiotics on barrier dysfunction and oxidative stress in experimental AP.

**Methodology/Principal Findings:**

Fifty-three male Spraque-Dawley rats were randomly allocated into five groups: 1) controls, non-operated, 2) sham-operated, 3) AP, 4) AP and probiotics and 5) AP and placebo. AP was induced by intraductal glycodeoxycholate infusion and intravenous cerulein (6 h). Daily probiotics or placebo were administered intragastrically, starting five days prior to AP. After cerulein infusion, ileal mucosa was collected for measurements of *E. coli* K12 and ^51^Cr-EDTA passage in Ussing chambers. Tight junction proteins were investigated by confocal immunofluorescence imaging. Ileal mucosal apoptosis, lipid peroxidation, and glutathione levels were determined and glutamate-cysteine-ligase activity and expression were quantified. AP-induced barrier dysfunction was characterized by epithelial cell apoptosis and alterations of tight junction proteins (i.e. disruption of occludin and claudin-1 and up-regulation of claudin-2) and correlated with lipid peroxidation (r>0.8). Probiotic pre-treatment diminished the AP-induced increase in *E. coli* passage (probiotics 57.4±33.5 *vs.* placebo 223.7±93.7 a.u.; *P*<0.001), ^51^Cr-EDTA flux (16.7±10.1 *vs.* 32.1±10.0 cm/s10^−6^; *P*<0.005), apoptosis, lipid peroxidation (0.42±0.13 *vs.* 1.62±0.53 pmol MDA/mg protein; *P*<0.001), and prevented tight junction protein disruption. AP-induced decline in glutathione was not only prevented (14.33±1.47 *vs.* 8.82±1.30 nmol/mg protein, *P*<0.001), but probiotics even increased mucosal glutathione compared with sham rats (14.33±1.47 *vs.* 10.70±1.74 nmol/mg protein, *P*<0.001). Glutamate-cysteine-ligase activity, which is rate-limiting in glutathione biosynthesis, was enhanced in probiotic pre-treated animals (probiotics 2.88±1.21 *vs.* placebo 1.94±0.55 nmol/min/mg protein; *P*<0.05) coinciding with an increase in mRNA expression of glutamate-cysteine-ligase catalytic (GCLc) and modifier (GCLm) subunits.

**Conclusions:**

Probiotic pre-treatment diminished AP-induced intestinal barrier dysfunction and prevented oxidative stress via mechanisms mainly involving mucosal glutathione biosynthesis.

## Introduction

Multi-organ-failure and systemic inflammatory response syndrome (SIRS) remain major causes of mortality at intensive care units [1]. There is compelling evidence for an important role of the gut in the origin and development of critical illness [2], [3]. Gut barrier dysfunction can propagate a pathophysiological state leading to increased mortality. Deitch *et al.* [4] demonstrated for example, that shock-induced intestinal hypoperfusion leads to release of reactive oxygen species (ROS) and oxidative stress resulting in barrier failure and release of pro-inflammatory mediators, enhancing a subsequent SIRS. Evidence suggests that ROS disrupt epithelial tight junctions (TJs) 5, 6 leading to barrier dysfunction [7]. Furthermore, ROS cause epithelial cell apoptosis [8] contributing to mucosal barrier failure [9]–[11] and associated mortality [12], [13] in experimental studies. Moreover, clinical evidence shows that increased intestinal apoptosis is a prominent event in patients who succumb from sepsis [14]. The mucosal barrier may be further compromised by overgrowth of enteric pathogens e.g. *Escherichia coli* [15] or by other opportunistic pathogens which switch on their virulence genes upon intestinal hypoxia [16], suggesting an important role for intestinal microbiota in gut-derived sepsis [2]. Taken together in critically ill patients, SIRS may be driven by an oxidative stress-induced disruption of the equilibrium of the otherwise symbiotic three-way partnership between intestinal microbiota, epithelium, and immune system.

Conversely, a moderate increase in intracellular ROS concentrations may paradoxically afford protection against oxidative stress *via* upregulation of oxidative defense mechanisms. Indeed, *de novo* synthesis of the most important endogenous antioxidant, glutathione (GSH) is found to be enhanced after low dose H_2_O_2_ [17] and is also increased by other weak oxidative agents [18].

Severe acute pancreatitis (AP), which is characterized by intestinal barrier dysfunction and not seldom leading to SIRS, represents a clinical disease in which maintenance of this equilibrium is severely disturbed [3]. Since commensal bacteria are believed to be a crucial part of host homeostasis, recent studies have looked at effects of probiotics in recreating equilibrium [19]–[21]. Our group previously developed a probiotic combination designed to prevent infectious complications in critical illness based on anti-inflammatory and microbiota modulating capacities [22]. Five-day pre-treatment with these multispecies probiotics attenuated bacterial translocation and reduced the mortality in experimental AP in rats [23], but recently we also demonstrated in a double-blind clinical study that these probiotics, contrary to any expectations, doubled the mortality compared with placebo in 298 patients with predicted severe AP [24]. These results painfully showed the need to study mechanisms of action of probiotics in critical illnesses. The objective of this study was to characterize the intestinal mucosal barrier in experimental AP and to explore mechanisms by which multispecies probiotics affect barrier function under these circumstances. We found that probiotics maintained the mucosal barrier in AP by up-regulation of the rate-limiting step in glutathione (GSH) biosynthesis.

## Materials and Methods

### Rats

**Figure 1:**
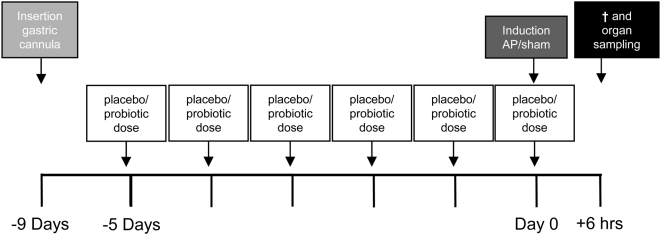
Experimental design. At the start of the experiment, animals were fitted with a gastric cannula, except for control animals. Probiotics and placebo were administered daily to the probiotics and placebo group, starting 5 days prior to induction of acute pancreatitis (AP). At day 0, AP or sham-procedure was performed. After the six hours of cerulein infusion, animals were anesthetized for removal of organ samples.

Male specific pathogen-free Sprague-Dawley rats (B&K, Sollentuna, Sweden, 250–350 g, 50–70 days of age) were maintained under constant conditions with a 12-hour light/dark cycle and free access to water and standard rat pellets. Rats were acclimatized for one week prior to surgery and randomly allocated into five groups: 1) non-operated controls (n = 5); 2) sham-procedure (n = 12); 3) AP (n = 12); 4) AP, placebo (n = 12); 5) AP, probiotics (n = 12). The experimental design (fig 1) was in accordance with guidelines of the Linköping University Animal Welfare Committee, following European legislation (2003/65/EC).

### Probiotics

The multispecies probiotics consisted of six viable, freeze-dried strains: *Lactobacillus acidophilus* (W70), *Lactobacillus casei* (W56), *Lactobacillus salivarius* (W24), *Lactococcus lactis* (W58), *Bifidobacterium bifidum* (W23), and *Bifidobacterium lactis* (W52) (previously classified as *Bifidobacterium infantis*) (Ecologic® 641, Winclove Bio Industries, Amsterdam, the Netherlands). Placebo, that consisted of the carrier of the probiotic product, i.e. cornstarch and maltodextran, was packed in identical coded sachets to guarantee blinding. Before daily administration, probiotic or placebo formulas were reconstituted in sterile water, for 15 min at 37°C. A single probiotic dose of 1.0 ml contained 5×10^9^ colony forming units (CFU) of bacteria.

### Surgical procedures

Under general anesthesia (2% isoflurane), a permanent gastric cannula was fitted in all rats, as performed previously [23]. Animals were allowed to recover for four days prior to the start of daily probiotics or placebo administrations through the cannula and then after five days of pre-treatment AP was induced as described by Schmidt *et al*. [25]. Briefly, pressure controlled (35<mmHg) retrograde infusion of 0.5 ml sterilized glycodeoxycholic acid (Sigma-Aldrich, Zwijndrecht, the Netherlands) into the biliopancreatic duct was followed by intravenous cerulein (5 µg/kg/h, 1 ml/h, for 6 h, Sigma-Aldrich). During the sham procedure, cannulation of the biliopancreatic duct without infusion of glycodeoxycholic acid was followed by intravenous saline (1 ml/h, 6 h). Three animals needed to be excluded due to detachment of the gastric cannula and two due to anaesthesiological failures.

### Collection of samples

Whole blood was sampled by tail vein puncture, before start of treatment and before induction of acute pancreatitis. After cerulein or saline infusion, rats were anaesthetized and 15 cm distal ileum, the pancreatic tail and whole blood were collected. Ten cm of ileum was used for Ussing chamber experiments and immediately submersed into ice-cold oxygenated Kreb's buffer (115 mM NaCl, 1.25 mM CaCl_2_, 1.2 mM MgCl_2_, 2 mM KH_2_PO_4_, and 25 mM NaHCO_3_, pH 7.35). The remainder was flushed with cold Kreb's buffer to remove adherent bacteria, stripped of the external muscle, freeze-dried and stored at −70°C until analyzed. Histological assessment verified that no bacteria remained associated with the tissue samples. Samples for histological and immunohistochemical examinations were formalin fixed, and embedded in optimum cutting temperature compound (Histolab, Västra Frölunda, Sweden). All analyses were run in duplicates.

### Ussing chamber experiments

Mucosal permeability was measured as previously described [26]. Briefly, ileum, stripped of external muscle while immersed in Kreb's buffer, was mounted into Ussing chambers (Harvard Apparatus Inc., Holliston, MA, USA [27]) where 9.6 mm^2^ tissue was exposed to 3 ml (1.5 ml each half-chamber) circulating, oxygenated Kreb's solution at 37°C. The serosal buffer contained 10 mM glucose as energy source and was osmotically balanced by 10 mM mannitol in the mucosal buffer. Chambers contained agar-salt bridges to monitor potential difference across the tissue for vitality assessment. Baseline values for short circuit current (Isc), indicating net ion secretion, and conductance (passive ion flux), were recorded at equilibrium, 40 min after mounting.

Transepithelial transport of macromolecules was assessed by measuring horseradish peroxidase (HRP) (Sigma-Aldrich), as model antigen, and ^51^Cr-EDTA (Perkin-Elmer, Boston, MA, USA) flux, as paracellular probe. HRP and ^51^Cr-EDTA were added to the mucosal side to a final concentration of 10^−5^ M and 34 µC_i_/ml, respectively. Serosal samples (300 µl) were collected at 0, 30, 60, 90 and 120 min after start and were used to analyze transepithelial fluxes of ^51^Cr-EDTA, expressed as cm/s·10^−6^, using a gamma-counter (1282 Compugamma, LKB, Bromma, Sweden). HRP-activity was determined as previously described [26], and transepithelial HRP flux was expressed as pmol·cm^−2^·h^−1^. Permeability was calculated in 3 ileal samples per rat during the 30–120 min period for both markers.

To assess bacterial passage, fluorescent *E.coli* K12 (1×10^8^ CFU/ml, Molecular Probes, Leiden, the Netherlands), killed by paraformaldehyde to stop reproduction without loss of antigenicity [28], were added after equilibration, to the mucosal side. After 120 min, the entire volume of serosal compartments was analyzed at 488 nm in a fluorimeter (Cary Eclipse, Varian, Victoria, Australia). One unit corresponds to 3.0·10^3^ CFU/ml [26].

### Immunohistochemistry

Frozen ileum sections (5 µm) of 4 rats per group were incubated with 5% bovine serum albumin, washed and incubated with a primary antibody (1∶50 rabbit anti-rat occludin, mouse anti-rat claudin-1 or mouse anti-rat claudin-2; Zymed Laboratories, San Francisco, CA, USA) for 1 h at room temperature. Following extensive washes, slides were incubated with Alexa Fluor®488 goat anti-mouse or anti-rabbit immunoglobulin-G (1∶500 dilution, Jackson ImmunoResearch Europe Ltd, London, United Kingdom) for 1 h at room temperature.

Apoptotic cells were detected by ‘in-situ cell death detection kit’ (Roche Diagnostics, Bromma, Sweden). Frozen ileum sections (5 µm) were permeabilized in 0.1 mol/l sodium citrate for 2 min on ice and incubated in terminal-deoxynucleotidyl-transferase-mediated-dUTP-nick-end-labeling (TUNEL) reaction mixture for 1 h at 37°C.

All sections were counterstained with 0.5 µM 4′,6-diamidineo-2-phenylindole (DAPI) for 10 min, mounted in antifading Fluorescent Mounting Medium (DakoCytomation, Stockholm, Sweden) and examined using confocal imaging with a 2-photon BioRad Radiance 2000 microscope (Carl Zeiss, Jena, Germany), equipped with high numerical aperture (NA = 1.4) 60× and 100× oil immersion objectives. Each test included negative controls. Image acquisition settings were identical for each experiment. Apoptotic rate was determined by counting the number of TUNEL^+^ cells/100 epithelial cells in 4 sections from 4 rats per group.

### DNA-fragmentation assay

Histone-associated DNA-fragmentation was determined in ileal homogenate corresponding to 50 µg freeze-dried mucosa as previously described [29], using Cell Death Detection ELISA ^PLUS^ (Roche Diagnostics). Results are normalized to protein content, as measured according to Bradford's method [30] and expressed as ratio to control animals.

### Histological measurements of mucosal damage

Coded ileal sections were haematoxylin-eosin (H&E) stained and the degree of mucosal damage was determined in 4 tissue sections per rat, by a pathologist blinded to the experimental design. Histopathological grading, from 0 (normal mucosa) to 5 (severe mucosal damage), was performed according to criteria by Chiu *et al.* [31].

To confirm pancreatitis, histological analysis of H&E stained pancreatic sections was performed in 4 tissue sections per rat, utilizing Spormann's criteria [32].

### Lipid peroxidation

To assess oxidative damage, malondialdehyde (MDA) concentration was determined, using a lipid peroxidation assay (LPO-586; Byoxitech, OXIS International, Portland, OR, USA). Ileal mucosa was homogenized in 5 mM butylated hydroxytoluene to prevent sample oxidation. Supernatants were used to determine MDA levels according to manufacturer's instructions. Results were normalized to protein contents of the crude homogenates.

### Glutathione assay

To estimate the antioxidative capacity, reduced and oxidized GSH contents were determined in ileal tissue and plasma using a commercially available assay (Glutathione Assay Kit II, Merck Chemicals, Hull, United Kingdom). To ensure absence of adherent bacteria, samples were flushed with cold Kreb's buffer, and microscopically examined. Freeze-dried ileal mucosa was homogenized in acid medium (0.2 M 2-(N-morpholino) ethanesulphonic acid, 0.05 M phosphate, and 1 mM EDTA, pH 6.0), centrifuged (10 min, 10,000×*g*) and supernatants were collected. After protein determination, supernatants corresponding with 1 mg protein, and plasma aliquots were deproteinized with 5% metaphosphoric acid (Sigma-Aldrich Chemie BV) and 4 M triethanolamine (Sigma-Aldrich Chemie BV) and plasma samples were lyophilized.

Individual bacterial strains from the used probiotics were grown in de Man-Rogosa-Sharpe (MRS) broth at 37°C for 24 h, under strict anaerobic conditions. To determine GSH release during bacterial growth, samples were taken at 0, 6, and 24 h, centrifuged (4,000×*g* for 10 min at 4°C) and supernatants were collected. For determination of bacterial GSH content, bacteria were collected after 24 h of cultivation and disrupted by sonication (Bransonic 3200, Branson Ultrasonics b.v., Soest, the Netherlands) on ice for 10 min with 3 sec cooling interval per min. Suspensions were centrifuged, yielding a cell-free extract.

Cell-free extracts, tissue supernatants and plasma samples were analyzed for total GSH according to the protocol provided by the manufacturer. To quantify oxidized GSH (GSSG), 2-vinylpyridine was added to the acidic medium to derivatize GSH. GSH levels were calculated by subtracting the amount of GSSG from the total GSH content and normalized to protein content.

### Cysteine

Mucosal cysteine was determined using the spectrophotometric method developed by Gaitonde [33] and expressed as nmol/mg protein.

### Glutamate-cysteine-ligase

Biosynthesis of GSH was analyzed by quantification of glutamate-cysteine-ligase (GCL, EC: 6.3.2.2) activity as previously described [34]. For determination of systemic GSH biosynthesis, erythrocytes were obtained by centrifugation of ETDA blood samples at 900×*g* for 3 min and after washing 3 times with 5 volumes of cold isotonic NaCl solution. Erythrocytes were lysed by the addition of 50 mmol Tris-HCl buffer (pH 7.4), containing 1 mmol EDTA, and by sonication for 2×20 seconds. The erythrocyte membranes were removed by centrifugation at 18,000×*g* for 40 min. For determination of local intestinal mucosal GCL activity, ileal tissues were homogenized in 250 mM sucrose containing 20 mM Tris, 1 mM EDTA, 20 mM boric acid, 2 mM serine, pH 7.4. GCL activity was determined as the difference between γ-glutamylcysteine (GC) synthesis in unblocked and GC synthesis in samples blocked with 200 mM 5-sulfosalicylic acid dehydrate and expressed as nmol (GC)/min/mg protein.

### mRNA expression analysis

Total RNA was isolated from ileal mucosa using the RNeasy Midi Kit (Qiagen, Hilden, Germany) and spectrophotometrically quantified, showing A260/A280 ratios within normal range. Subsequently, the integrity of total RNA was checked by denaturing agarose gel electrophoresis. First strand cDNA was synthesized from total RNA using the iScript cDNA synthesis kit (BioRad, Hercules, CA, USA) and quantitative RT-PCR was performed using the iCycler iQ system (BioRad). RT-PCR with mRNA-specific primers for the catalytic (GCLC) and modifier (GCLM) subunits of GCL and 18S rRNA as a reference gene was performed (GCLC-forward 5′-ggcgatgttcttgaaactctg-3′, GCLC-reverse 5′-cagagggttgggtggttg-3′; GCLM-forward 5′-ctgactcacaatgacccaaaag-3′, GCLM-reverse 5′-ttcaatgtcagggatgctttc-3′; 18S rRNA-forward 5′-aatcagttatggttcctttgtcg-3′, 18S rRNA-reverse 5′-gctctagaattaccacagttatccaa-3′; Sigma-Aldrich) and mRNA levels were quantified using SYBR Green based detection.

Prior to real-time PCR analysis cDNA samples were diluted 1∶25, except for 18S rRNA which was diluted 1∶1000, with RNase-free water. PCR reactions were set up in a volume of 25 µl, containing 5 µl of diluted cDNA, 12.5 µl of 2× iQ SYBR Green Supermix (BioRad) and 300 nM of the forward and reverse primer each. Thermal cycling conditions were 95°C for 3 min as initial denaturation and enzyme-activating step followed by 40 cycles of 95°C for 15 s denaturation, 60°C for 30 s annealing and 72°C for 30 s extension. After amplification a melting curve analysis was performed by increasing the temperature by 0.5°C increments from 55°C to 95°C and measuring fluorescence at each temperature for a period of 10 s. All cDNA samples were analyzed in triplicate and each run contained a relative standard curve. Purified PCR products were used to generate the relative standard curves, consisting of serial dilutions. Levels of GCLC and GCLM mRNA were quantified using the comparative threshold cycle method, normalized to18S rRNA expression and expressed as ratio to controls (a.u.).

### Statistical Analysis

Normal distribution was assessed using Shapiro–Wilk's test. Parametric values are presented as mean (SD). Statistical analysis was performed by ANOVA followed by Tukey's HSD test. Non-parametric values are given as median (25–75^th^ interquartile range). Comparisons between two groups were done by Mann-Whitney U-test and between multiple groups by Kruskal-Wallis. Spearman's rank correlation coefficients were computed for correlation analyses. Considering Bonferroni's correction, *P*<0.01 was considered significant.

## Results

### Acute pancreatitis induced severe ileal mucosal barrier dysfunction

Mortality due to AP did not occur. Pancreatitis was confirmed by histological scoring of pancreatic injury [32] (sham-operated 0 (0-0) *vs.* after induction of pancreatitis 3 (2–5.1); *P*<0.001).

**Figure 2:**
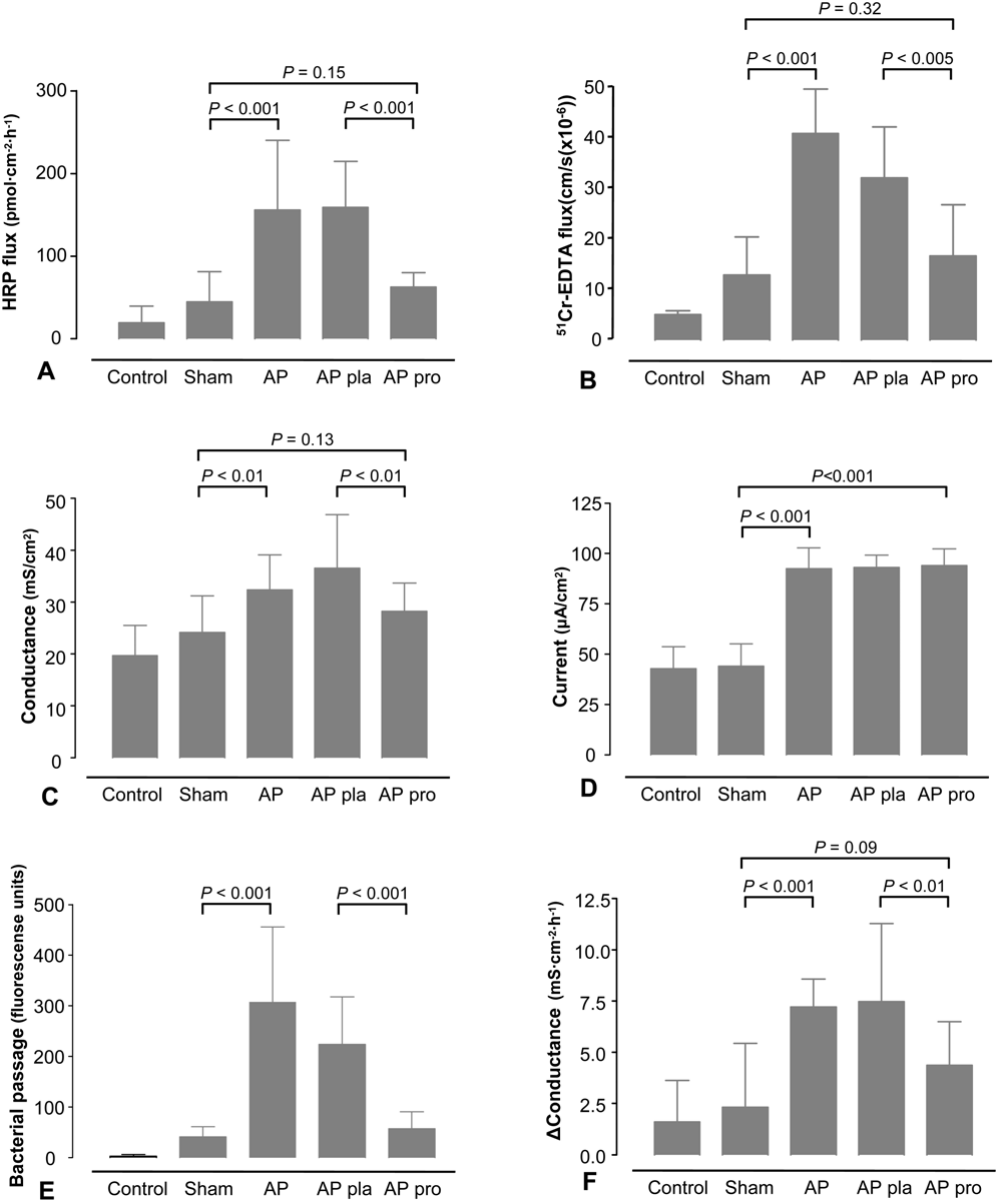
Probiotics prevented acute pancreatitis-induced ileal permeability but not ion secretion. After 5 days of pre-treatment with placebo (pla, n = 12) or probiotics (pro, n = 12), rats were subjected to acute pancreatitis (AP, n = 12), a sham-procedure (n = 12) or not operated (control, n = 5). Ileal segments were mounted in Ussing chambers and (A) horseradish peroxidase (HRP) and (B) ^51^Cr-EDTA flux were studied for two hours. (C) Baseline conductance, (D) baseline short circuit current (Isc), (E) passage of *Escherichia coli* K12 and (F) elevation of conductance during one hour after challenge with *E. coli* were measured. The graphs show average (±SD). The data were collected from independently acquired sets of 3 tissue segments per rat. Comparisons were performed using ANOVA followed by Tukey's HSD.

Pancreatitis induced increased permeability to HRP and ^51^Cr-EDTA (fig 2A, B), accompanied by increase in baseline conductance, representing paracellular ion flux (fig 2C) and elevation of Isc, indicative of ion secretion (fig 2D). Moreover, transepithelial bacterial passage increased by as much as 7-fold in animals subjected to AP (fig 2E) and tissues from rats in the pancreatitis group responded to *E. coli* K12 added to the luminal buffer with an enhanced elevation in conductance (fig 2F). These data suggest a combined perturbation of paracellular and transcellular pathways.

**Figure 3:**
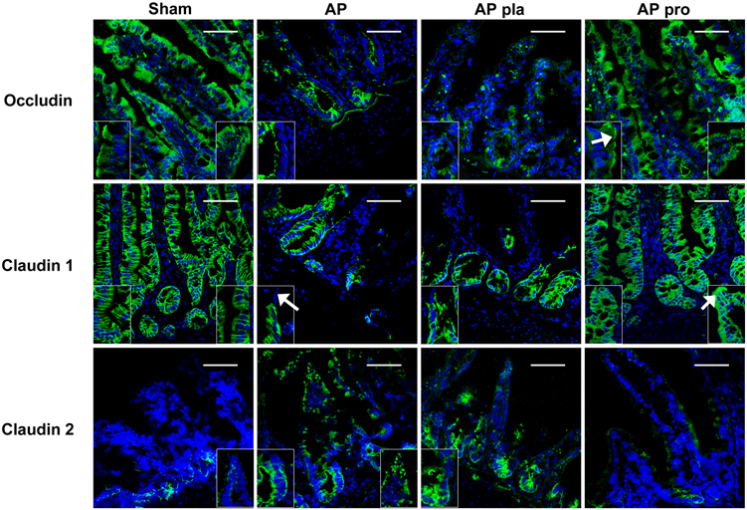
Probiotics prevented disruption of tight junction proteins. After 5 days of pre-treatment with placebo (pla) or probiotics (pro), rats were subjected to acute pancreatitis (AP), or a sham-procedure. Ileal sections were stained with occludin, claudin-1 or -2 antibodies (green), counterstained with DAPI (blue) and visualized by confocal laser scanning microscopy. Bar = 500 µm. The higher magnification (100×/1.30) images shown in the insets are typical details of crypts (left) and villi (right). Probiotics prevented the deleterious effects of AP on occludin and caused redistribution of occludin to the apical surface (arrowhead). Acute pancreatitis-induced detachment of epithelial cells seems to be preceded by loss of claudin-1 (arrowhead) and was reduced by probiotic pre-treatment; though probiotics could not prevent the AP-induced formation of aggregates of claudin-1 in the cytosol (arrowhead). Probiotics prevented AP-associated up-regulation of claudin-2 in both crypts and villi (arrowhead). The patterns of staining are typical of that seen in 4 sections of 4 rats per group.

To further characterize the paracellular pathway TJ protein expression was studied in tissue sections. In sham-operated animals, occludin was localized in the cytoplasm of epithelial cells and along the basolateral membrane with an enrichment of occludin at the apical surface (fig 3). In AP partial disruption of occludin was seen in crypts as well as in villi. Claudin-1 staining pattern was diffuse with predominantly intracellular localization in sham-operated animals (fig 3). AP caused decreased staining intensity and aggregation of claudin-1 within the cytosol. Claudin-1 was not detected near areas of epithelial disruption, suggesting that detachment of enterocytes was preceded by loss of claudin-1. Contrary to occludin and claudin-1, the pore-forming TJ protein, claudin-2 [7], was only scarcely detectable in crypts of sham-operated animals, whereas rats from the AP group showed intense staining of claudin-2 both in crypt and surface epithelium (fig 3).

**Figure 4:**
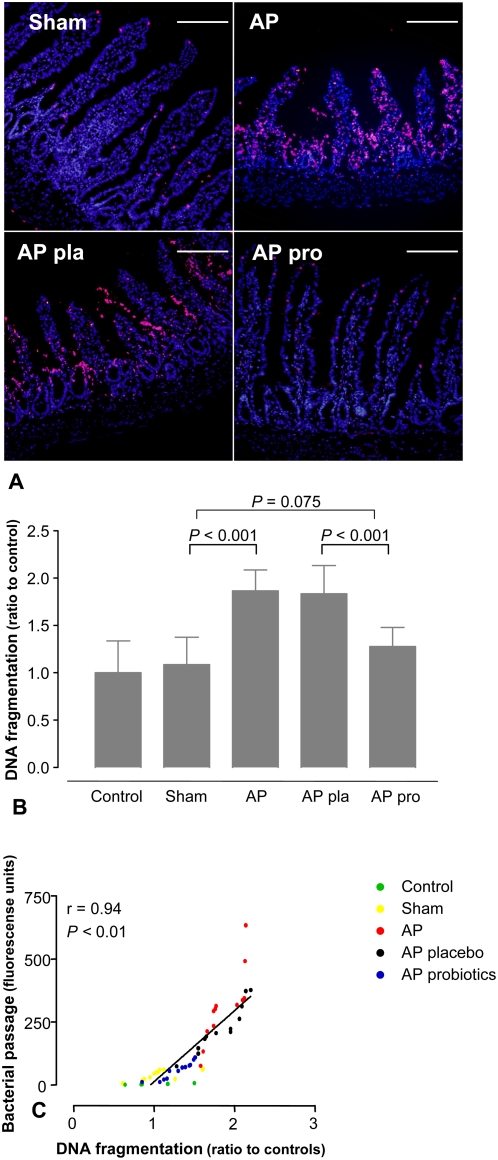
Probiotics reduced pancreatitis-associated intestinal apoptosis. After 5 days of pre-treatment with placebo or probiotics, rats were subjected to acute pancreatitis, or a sham-procedure. (A) Sections of ileum were TUNEL stained. The results shown are typical images from 4 sections of 4 rats per group. Bar = 200 µm. (B) DNA-fragmentation (control n = 5, sham n = 12, AP n = 12, AP pla n = 12 and AP pro n = 12). The graph shows the average (±SD). Comparisons were performed using ANOVA followed by Tukey's HSD. (C) Positive correlation between intestinal apoptosis and *Escherichia coli* K12 passage was computed using Spearman's rank correlation coefficients.

It is reported that intestinal epithelial apoptosis contributes to mucosal barrier dysfunction [9]–[11]. Confocal microscopy with immunofluorescent TUNEL staining (fig 4A) revealed AP-induced epithelial cell apoptosis compared to sham animals (25.8 (24.4–26.3) *vs.* 2.60 (2.47–2.73) TUNEL^+^cells/100 epithelial cells; *P*<0.001). Mucosal DNA-fragmentation corroborated these findings (fig 4B). Moreover, DNA-fragmentation strongly correlated with bacterial passage (fig 4C), ^51^Cr-EDTA flux (r = 0.93) and tissue conductance (r = 0.87), which supports the hypothesis that epithelial cell apoptosis disrupts barrier integrity.

**Figure 5:**
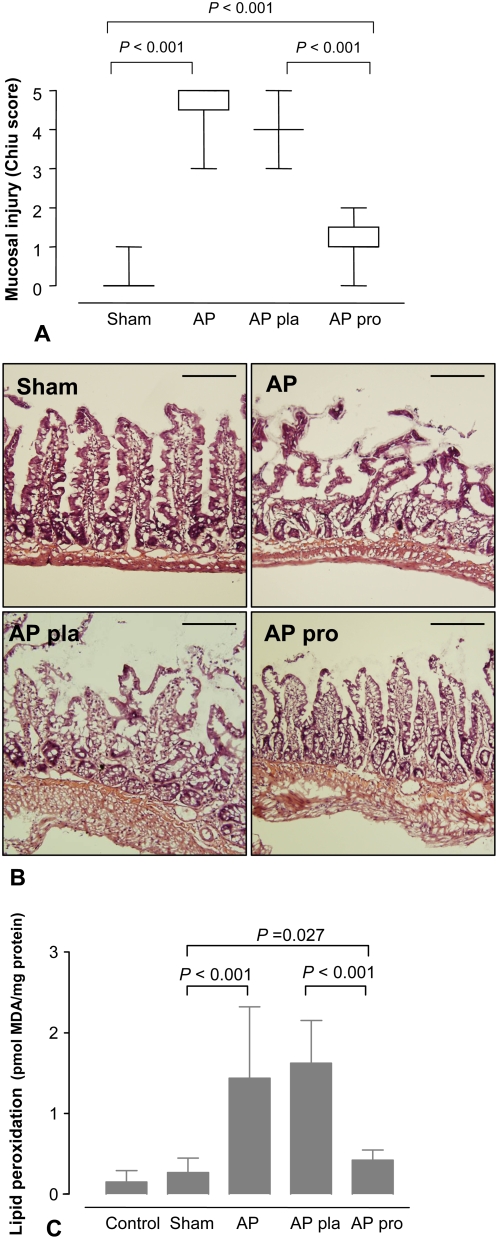
Probiotics attenuated acute pancreatitis-associated mucosal damage. After 5 days of pre-treatment with placebo (pla, n = 12) or probiotics (pro, n = 12), rats were subjected to acute pancreatitis (AP, n = 12), or a sham-procedure (n = 12). (A) Sections of ileum were H&E stained and graded according to Chiu *et al.* (25). The graph shows median (±range). Comparisons were performed using Kruskal-Wallis followed by Mann–Whitney *U* test. (B) Compared with sham-operated animals (Sham), acute pancreatitis (AP) caused widespread destruction of villi. Placebo treated animals (AP pla) also showed a severe degree of mucosal damage. Probiotic animals (AP pro) showed extensive epithelial lifting, but with intact epithelium. The mucosal damage is typical of that seen in 4 sections from 12 rats per group. Bar = 200 µm. (C) Lipid peroxidation (MDA levels) (control n = 5, sham n = 12, AP n = 12, AP pla n = 12 and AP pro n = 12). The graph shows the average (±SD). Comparisons were performed using ANOVA followed by Tukey's HSD.

Six hours after induction of pancreatitis, intestinal injury (fig 5A) was characterized by villus denudation, lamina propria disintegration and ulceration (fig 5B), resembling intestinal ischemia-reperfusion injury [31], which is associated with ROS release, epithelial apoptosis and TJ disruption [5], [6], [8]. Therefore, oxidative stress-induced lipid peroxidation was quantified, which was indeed found to be elevated after induction of AP (fig 5C) and also showed a strong positive correlation with barrier dysfunction (^51^Cr-EDTA flux r = 0.83, bacterial passage r = 0.88).

### Probiotics prevented acute pancreatitis-induced barrier dysfunction

No rats receiving probiotics showed signs of diarrhea or loss of appetite during the pre-treatment period. Increase in animal weight was similar in all groups (sham-operated 22.3 (1.09) *vs.* AP 21.9 (1.11) *vs.* placebo 20.6 (0.82) *vs.* probiotics 20.5 (0.61)). Five days of pre-treatment with probiotics abolished the deleterious effects of AP on numerous parameters of barrier function. AP-induced increase in ileal permeability to HRP (fig 2A) and ^51^Cr-EDTA (fig 2B) as well as tissue conductance (fig 2C) was normalized after probiotic pre-treatment. Elevation in tissue conductance after adding *E.coli* K12 was 40% smaller in tissues from probiotic treated rats compared to placebo (fig 2F). In contrast, there were no inhibitory effects of probiotics on AP-induced elevation of Isc (fig 2D).

Probiotics also modified the localization of TJ proteins. AP-associated partial disruption of the distribution of occludin in crypts and villi was prevented and redistribution to the apical surface was apparent in crypts of probiotic treated animals (fig 3). In both claudin-1 and -2 staining patterns the AP-induced deleterious effects were reduced by probiotic pre-treatment (fig 3).

Furthermore, probiotics attenuated AP-induced epithelial cell apoptosis, showing a 70% reduction in apoptotic rate (8.85 (8.60–9.15) *vs.* placebo 31.75 (31.60–32.90) TUNEL^+^ cells/100 epithelial cells; *P*<0.001, fig 4A), which was also demonstrated by analysis of mucosal DNA-fragmentation (fig 4B). Histological scoring demonstrated that probiotics ameliorated pancreatitis-induced mucosal damage (1.0 (1.0–1.25) *vs.* placebo 4.0 (4.0-4.0); *P*<0.001, fig 5A) and normalized mucosal lipid peroxidation (fig 5C).

### Beneficial effect of probiotics by increasing mucosal glutathione

**Figure 6:**
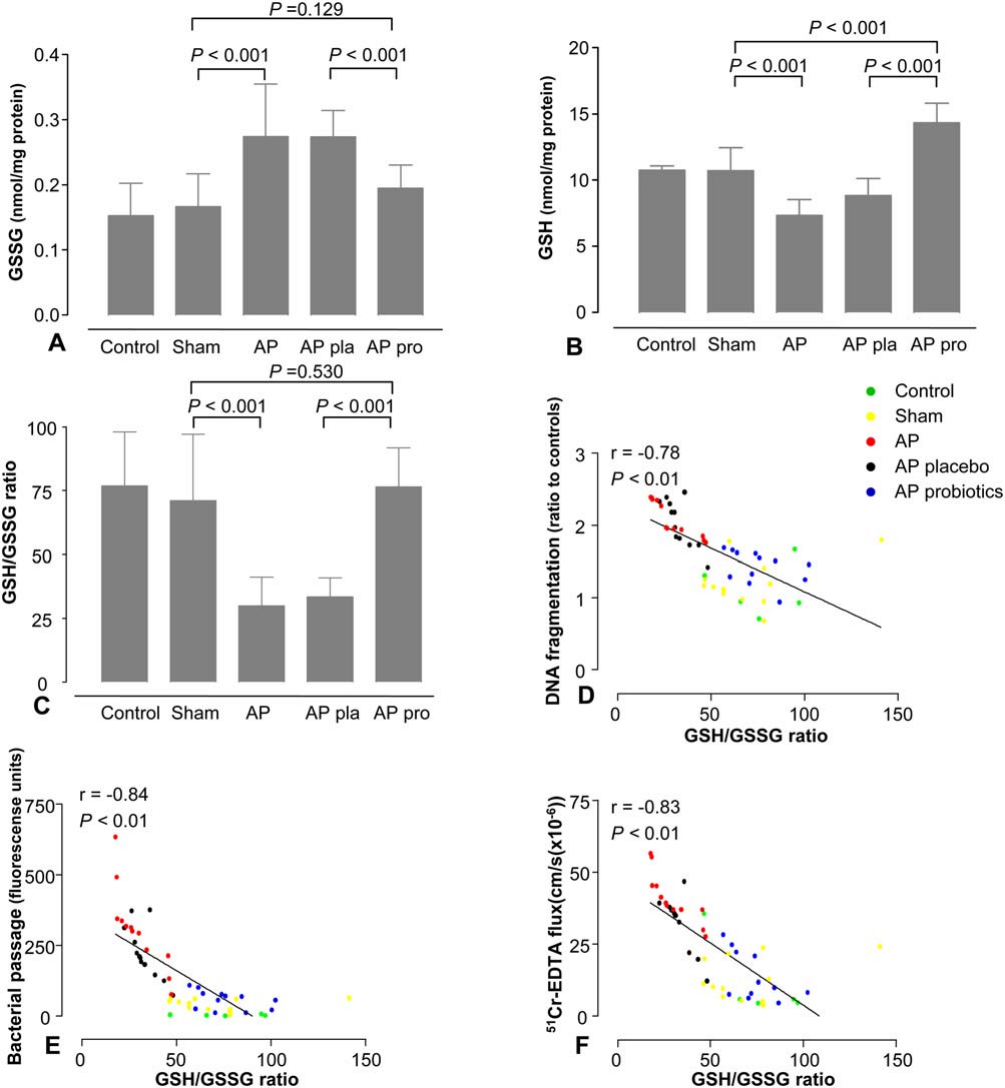
Probiotics enhanced mucosal glutathione levels. After 5 days of pre-treatment with placebo (pla, n = 12) or probiotics (pro, n = 12), rats were subjected to acute pancreatitis (AP, n = 12), a sham-procedure (n = 12) or not operated (control, n = 5). Six hours after induction of the AP or sham-procedure, mucosal (A) oxidized glutathione levels (GSSG), (B) reduced glutathione levels (GSH) and (C) GSH/GSSG ratios were determined. The graphs show average (±SD). Comparisons were performed using ANOVA followed by Tukey's HSD. Correlation analyses revealed an inverse correlation between GSH/GSSG ratio and (D) DNA-fragmentation, (E) ileal permeability to *Escherichia coli* K12 and (F) ^51^Cr-EDTA flux. Spearman's rank correlation coefficients were computed for correlation analyses.

The decline in mucosal lipid peroxidation after probiotic pre-treatment may have resulted from either reduced amounts of ROS, or enhanced antioxidative capacity. Therefore, we quantified mucosal oxidized glutathione (GSSG) and GSH in thoroughly rinsed ileal mucosal tissues. Probiotics attenuated AP-induced elevation in GSSG (fig 6A), prevented depletion of mucosal GSH (fig 6B) and normalized the GSH/GSSG ratio (fig 6C). Of note, mucosal GSH/GSSG ratios showed an inverse correlation with DNA-fragmentation (fig 6D) and mucosal barrier dysfunction (fig 6E, F). Most interestingly, pre-treatment with probiotics induced increased levels of GSH, also in comparison with healthy control animals (fig 6B).

### Production of glutathione by the individual probiotic strains

Since mucosal GSH is partially dependent on uptake of dietary GSH [35], we quantified intrabacterial GSH of the separate probiotic strains, as well as GSH levels in medium after 6 and 24 hours of strictly anaerobic cultivation. Only *B. bifidum*, *B. lactis* and *L. acidophilus* contained abundant intracellular GSH (Table 1). GSH in cultivation medium increased over time, except for *Lc. lactis*.

**Table 1:** Intracellular GSH contents of the probiotic strains and GSH levels in medium at different time points after start of cultivation.

Bacterial strain	Intracellular GSH (nmol/mg protein)	GSH content in culture medium (nmol/ml supernatant)		
		0 hour	6 hours	24 hours
*B. bifidum* W23	0.37 (0.002)	0.00 (0.000)	0.10 (0.006)	0.51 (0.006)
*L. salivarius* W24	0.11 (0.002)	0.00 (0.000)	0.08 (0.004)	0.15 (0.006)
*B. lactis* W52	0.01 (0.002)	0.00 (0.000)	0.00 (0.000)	0.01 (0.008)
*L. casei* W56	0.09 (0.004)	0.00 (0.000)	0.00 (0.000)	0.01 (0.002)
*Lc. lactis* W58	0.04 (0.014)	0.00 (0.000)	0.00 (0.000)	0.00 (0.000)
*L. acidophilus W70*	0.14 (0.005)	0.00 (0.000)	0.04 (0.002)	0.19 (0.002)

*Bifidobacterium (B.)*; *Lactobacillus (L.)*; *Lactococcus (Lc.)*; glutathione (GSH).

Mean (SD), n = 4 separate experiments.

### Local mucosal biosynthesis of glutathione

**Figure 7:**
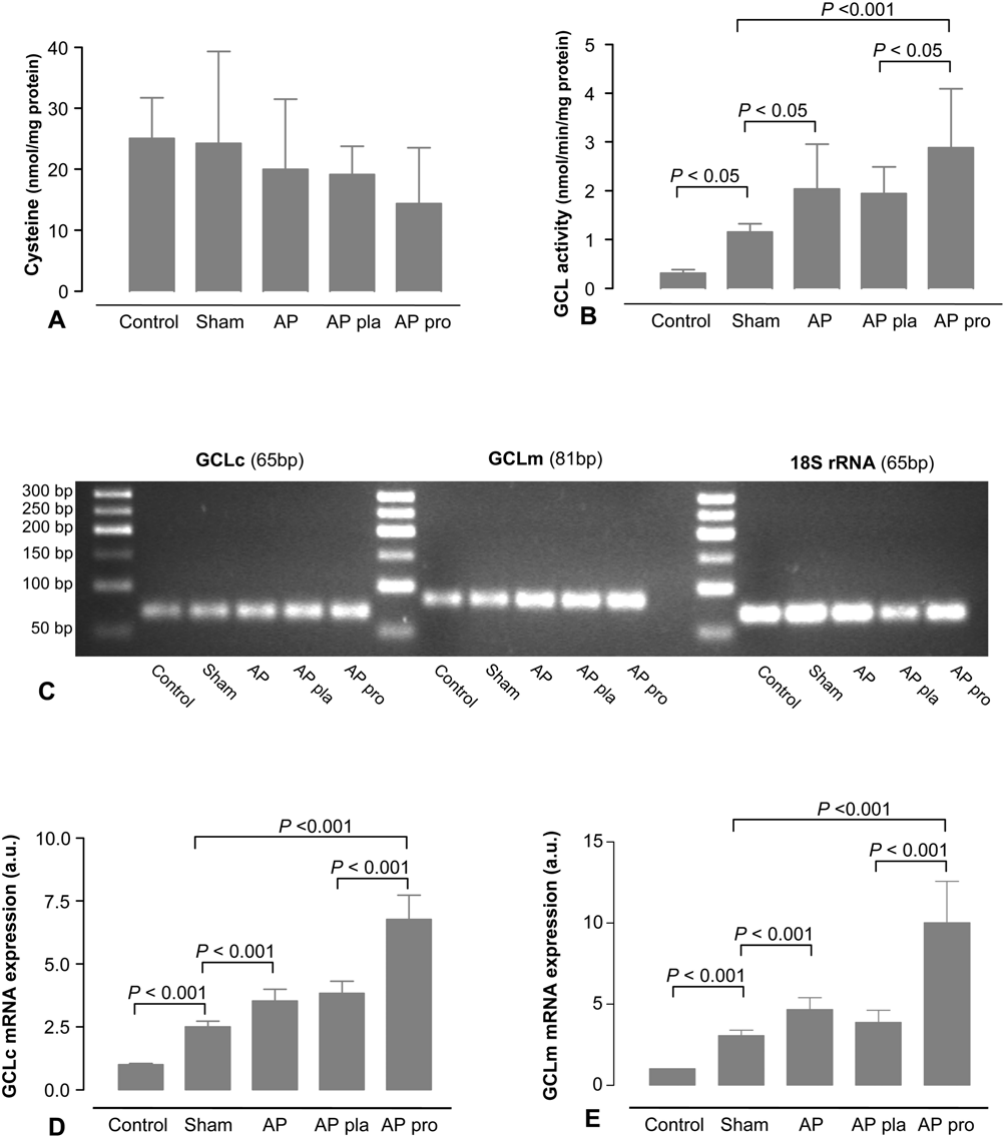
Probiotics have no effect on cysteine availability, but induce glutamate-cysteine-ligase activity. After 5 days of pre-treatment with placebo (pla, n = 12) or probiotics (pro, n = 12), rats were subjected to acute pancreatitis (AP, n = 12), a sham-procedure (n = 12) or not operated (control, n = 5). Six hours after induction of the AP or sham-procedure, tissue cysteine availability (A) and mucosal glutamate-cysteine-ligase (GCL) activity (B) were determined in ileum samples. (C) RT-PCR was conducted on ileal mRNA. PCR products of specific primers for the catalytic (GCLc, 65 bp) and the modulatory (GCLm, 81 bp) subunit of GCL and for 18S rRNA (65 bp) as control were identified on 2.5% agarose gel, using a GeneRuler 50 bp DNA Ladder (Fermentas GMBH, St. Leon-Rot, Germany). mRNA expression of (D) GCLc and (E) GCLm were quantified. Data are normalized to 18S rRNA expression and expressed as ratio to control animals. The graphs show average (±SD). All analyses were run in triplicates. Comparisons were performed using ANOVA followed by Tukey's HSD.

Mucosal GSH is, besides dietary uptake, also dependent on biosynthesis, which is regulated by availability of cysteine and GCL activity [36]. Mucosal cysteine levels did not differ significantly between the groups (fig 7A). GCL activity, however, was affected by AP and by probiotics pre-treatment. AP *per se* increased GCL activity compared to sham, but the most abundant increase was seen in the probiotics group which showed a 10-fold increase compared to controls (fig 7B).

Because GCL is composed of both a catalytic and modulatory subunit [37], the increase in GCL activity in this study could be due to enhanced expression of either GCLm and/or GCLc. Therefore, quantitative real-time PCR was performed to monitor changes in GCLc and GCLm message abundance. The level of mRNA expression of the reference gene, 18S, was comparable between all groups. When normalized to 18S, GCLc levels in probiotic pre-treated rats were 6.78 (0.95) a.u., which was 1.8 fold higher than in placebo treated rats (3.83 (0.49) a.u., fig 7C, D). Similarly, levels of GCLm mRNA after probiotic pre-treatment (10.3 (2.56) a.u) were 2.7 fold higher than average values seen in placebo treated rats (3.88 (0.75) a.u). The increase in GCLm mRNA expression was less pronounced in the AP and placebo groups when compared to sham operated animals (3.07 *vs.* AP 4.68 a.u.; 3.07 *vs.* placebo 3.88 a.u., respectively, fig 7C, E). These data suggest that the enhanced GCL activity after probiotic pre-treatment may be due to increased gene expression in the ileal mucosa.

### Probiotics increase systemic glutathione levels

**Figure 8:**
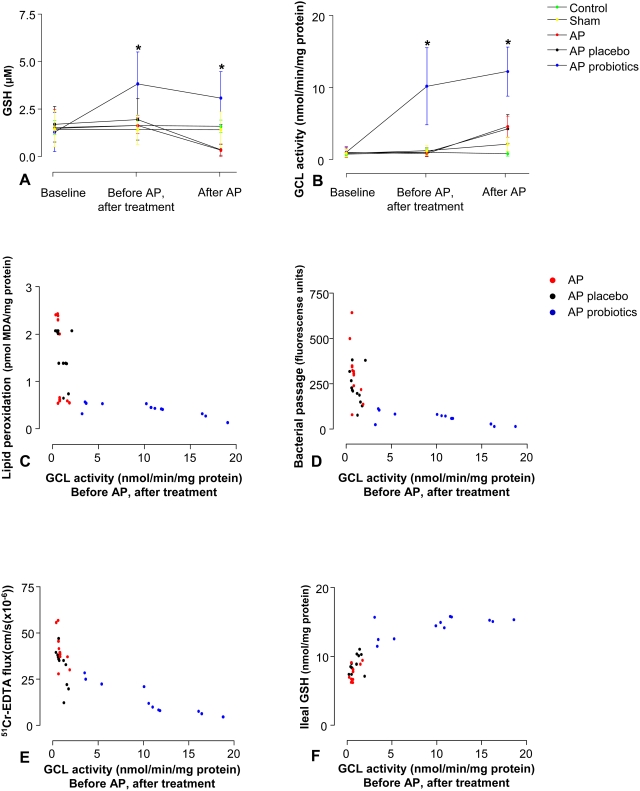
Probiotics induce systemic increase in GSH levels and GCL activity. After 5 days of pre-treatment with placebo (pla, n = 12) or probiotics (pro, n = 12), rats were subjected to acute pancreatitis (AP, n = 12), a sham-procedure (n = 12) or not operated (control, n = 5). Whole blood was sampled 1) before treatment, 2) after 5 days of pre-treatment, immediately before induction of AP and 3) six hours after induction of AP or sham-procedure. Time course of plasma GSH levels (A) and GCL activity in red blood cells (B) was monitored. The graphs show average (±SD). All analyses were run in duplicates. Comparisons were performed using ANOVA followed by Tukey's HSD. *P<0.001, probiotics *vs.* placebo. Associations between (C) ileal lipid peroxidation, (D) bacterial passage, (E) ^51^Cr-EDTA flux, (F) ileal GSH content six hours after induction of AP and GCL activity in red blood cells immediately before subjection to AP.

To gain insight into the antioxidative capacity prior to induction of AP, plasma GSH levels before treatment, before induction of AP and at time of termination were determined. In the course of the 5 days of the pre-treatment period, plasma GSH levels showed a 2 fold increase in probiotic pre-treated animals (fig 8A). This was in contrast to rats receiving placebo, in which plasma GSH levels did not differ significantly after 5 days of pre-treatment. This is in keeping with the increase in GCL activity in red blood cells (RBC) after 5 days of pre-treatment with probiotics (fig 8B). Correlation analyses between pancreatitis-induced oxidative damage, as measured by ileal lipid peroxidation and GCL activity immediately before induction of pancreatitis, suggested that GCL activity greater than 5 nmol/min/mg protein was protective against oxidative injury (fig 8C). Furthermore, GCL activity in RBCs immediately before induction of acute pancreatitis correlated inversely with parameters of mucosal barrier dysfunction in animals subjected to AP (bacterial passage: r = −0.80, ^51^Cr-EDTA: r = −0.86, fig 8D, E). Not surprisingly, GCL activity also correlated positively with ileal GSH levels in animals subjected to AP (r = 0.82, fig 8F).

## Discussion

The present study is the first to demonstrate that pre-treatment with multispecies probiotics increases mucosal GSH levels and stimulates GSH biosynthesis in the ileum, resulting in attenuated oxidative mucosal damage. Furthermore, normalization of GSH/GSSG ratios strongly correlated with improved barrier function. Therefore, increased mucosal GSH levels represent a candidate mechanism underlying the protection against barrier dysfunction afforded by pre-treatment with probiotics in experimental AP.

GSH synthesis was up-regulated in probiotic pre-treated rats, as demonstrated by enhanced GCL activity and increased mRNA expression of both of the GCL subunits, shown herein. GSH plays a pivotal role in maintenance of the redox balance (expressed as GSH/GSSG ratios), preventing oxidative damage [4] and maintaining mucosal barrier, which was reflected by the inverse correlation between mucosal GSH/GSSG ratios and parameters of barrier dysfunction. Two factors directly associate with mucosal GSH: dietary GSH levels [35] and GSH biosynthesis, of which the latter is in turn dependent on cysteine availability and GCL activity [36]. First, it has been reported that certain probiotics contain and release GSH [20], [38], [39] and Peran *et al.* [20] previously showed increased intestinal GSH following oral administration of *Lactobacillus fermentum* in experimental colitis. Our present *in vitro* experiments showed strain specific differences in intracellular GSH content within the range previously reported [38]. Moreover, time-dependent GSH release was found during anaerobic cultivation, which was abundant in *B. bifidum*, *B. lactis* and *L. acidophilus*. Nevertheless, considering an estimated GSH production of 31.0 nmol GSH by the total administered probiotic dose (17.9 nmol intrabacterial GSH (mean 0.13 nmol GSH/mg protein) +13.1 (1.53) nmol GSH secreted in 5 days; calculated from Table 1) compared with an estimated total increase in small intestinal GSH of 1190 nmol (small intestinal length 90 cm, mucosal protein content 2.4 mg/cm (n = 6); pre-treatment yielded increase in ileal GSH of 5.5 nmol/mg protein; placebo 8.8 *vs.* probiotics 14.3 nmol/mg protein, fig 6B), bacterial GSH could only partially account for the rise in ileal GSH content. Consequently, the possibility of local GSH biosynthesis was investigated. The present study did not show significant differences in mucosal cysteine, implying that cysteine availability was not an important discriminating factor. On the other hand, we found enhanced GCL activity and expression of the GCL subunits GCLc and GCLm in probiotic-treated animals leading to a significant increase in GSH contents. Although the contribution from the probiotic bacteria may be higher than calculated because of colonization and expansion, it is conceivable that enhanced CGL activity in the intestinal mucosa was the major factor contributing to the increased ileal GSH content.

Interestingly, correlation analysis between GCL activity and parameters of mucosal barrier failure in animals subjected to AP, suggested the existence of a threshold GCL activity above which mucosal protection against oxidative stress is functional. This may explain that the relatively small increase in GCL activity between the probiotic and the placebo pre-treated groups resulted in considerable protection, whereas the rise in GCL activity between the sham and the AP group did not ameliorate the AP-induced damage. This hypothesis is supported by the correlation between GCL activity and ileal GSH content; the latter was only above a certain threshold of GCL activity able to withstand the deleterious effects of AP.

As previous experimental studies have shown that GCL gene expression is up-regulated both after low dose H_2_O_2_ [17] and after administration of weak inducers of oxidative stress [18], the increase in GCL activity found in the present study could be indicative of cellular stress as a mechanistic factor. Administration of probiotics may have caused a minor oxidative assault, e.g. intracellular accumulation of short-chain fatty acids produced by the bacteria, thereby inducing increased capacity of antioxidant enzymes, preconditioning the mucosa for a major oxidative attack during AP. This hypothesis is further supported by the found increase in systemic GCL activity in the probiotic pre-treated group, which was markedly enhanced already before the induction of AP. At first glance, it may seem contradictory to the current study that the recent placebo-controlled trial by Besselink *et al.* [24], demonstrated increased incidence of bowel ischemia after administration of probiotics in the acute phase of severe AP. However, keeping in mind that enteral probiotics caused low dose oxidative stress, probiotics administered after the onset of AP may act as an extra oxidative burden in an already critically affected redox system [3] thereby, causing increased oxidative stress-induced damage and ischemia.

During critical illness oxidative stress disrupts TJs [5], [6], which are crucial in determining epithelial barrier properties [7], as illustrated here by the AP-induced breach in barrier function. Disruption of TJs results in increased permeability to luminal antigens and bacteria that promote release of pro-inflammatory cytokines which further deteriorates mucosal barrier function [2]. This is in keeping with our results that are the first to show AP-induced disruption of the claudin-1 distribution together with up-regulation of claudin-2, which is also the case in inflammatory bowel disease and destabilizes TJs [7], [10], [40]. Immunostaining revealed that pre-treatment with probiotics maintained TJ integrity with a normal distribution of claudin-1 and -2. The finding of Yasuda and colleagues [9], that AP did not have deleterious effects on occludin, is in contrast with our results. This may, however, be explained by differences in the model of AP used.

Apoptosis is the major mode of cell death during intestinal ischemia/reperfusion [8] and exerts deleterious effects on mucosal barrier function and survival [9]–[11], [13], [14]. Yan *et al.* [41] previously showed that soluble proteins produced by *Lactobacillus* strains protect epithelial cells from cytokine-induced apoptosis. Here, we found that probiotics, which induced GSH biosynthesis, normalized AP-induced epithelial cell apoptosis. In addition, we were able to demonstrate a positive correlation between mucosal DNA-fragmentation and barrier dysfunction, providing further evidence that oxidative stress plays an important role in induction of epithelial apoptosis and subsequent loss of barrier function.

Contrary to the effects on permeability, pre-treatment with probiotics showed no effect on ion secretion in our experiment. These findings emphasize the divergent regulation of cellular secretory and barrier functions. It has been reported that epithelia respond rapidly to pathogenic bacteria with altered ion secretion [42], indicating that “flushing out” may be a defense mechanism against the threat of invasion of the mucosa. In this regard, it is perhaps advantageous that probiotics do not inhibit these beneficial adaptive responses to an infectious threat.

In conclusion, the present study is to our knowledge the first to show that pre-treatment with multispecies probiotics, stimulates mucosal GSH biosynthesis and consequently normalizes AP-induced barrier dysfunction and attenuates epithelial cell apoptosis and disruption of TJs in a model of AP. In addition, our data demonstrate strong inverse correlations between mucosal GSH/GSSG ratios and mucosal barrier dysfunction. This further supports the functional relevance of this endogenous antioxidant and gives novel insights into the mechanisms of probiotics. However, as the used compound is a multispecies combination of probiotic strains, it is worth noting that the found effects depend on the combination of the applied bacteria. Additional studies will be necessary to elucidate the effects of each separate strain as well as possible synergistic effects of this specific combination of probiotics.

In addition, the role of oxidative stress has been evaluated in experimental models of acute pancreatitis [43] and it should be emphasized that oxidative stress and excessive ROS generation are early features in AP and consequently a difficult target for clinical prophylaxis to prevent a severe course of the disease. This has recently been shown in a randomized controlled trial, utilizing intravenous antioxidant (n-acetylcysteine, selenium, vitamin C) therapy, where the results in AP patients were not that encouraging [44]. However, oxidative stress is not only involved in the early stage of AP, but also in the course of the disease and may for that reason be a target for therapy at later stages of the disease. However, as the probiotics used in the current study showed severe adverse effects in intensive care AP patients [24], and since the present effects on GCL activity most likely resulted from a mild oxidative stress, this combination of probiotics is not a defendable treatment option in critically ill patients. Therefore, the appropriate clinical use of multispecies probiotics would be a preventive approach to improve defense against an expected oxidative attack, such as before elective major abdominal surgery [45] or maintenance treatment in IBD and pouchitis [46], [47].
